# Cost-effectiveness of short implants (6–8.5 mm) compared to regular length implants (> 10 mm) with bone regeneration in posterior atrophic mandible: a 8-year microsimulation model

**DOI:** 10.1186/s12903-026-09152-2

**Published:** 2026-07-10

**Authors:** Gustavo Sáenz-Ravello, Mauricio Baeza, Shengchi Fan, Cristina De-la-Rosa-Gay, Eduard Valmaseda-Castellón, Nikos Mattheos

**Affiliations:** 1 Center for Epidemiology and Surveillance of Oral Diseases (CESOD), Faculty of Dentistry, University of Chile, Santiago, Chile; 2 Department of Conservative Dentistry, Faculty of Dentistry, University of Chile, Santiago, Chile; 3 Faculty of Dentistry, Chulalongkorn University, Bangkok, Thailand; 4 Department of Oral and Maxillofacial Surgery, Plastic Surgery, University Medical Center Mainz, Mainz, Germany; 5 Oral Surgery and Implantology, Faculty of Medicine and Health Sciences, University of Barcelona, Barcelona, Spain; 6 Department of Dentistry, Faculty of Medicine and Health Sciences, Universitat de Barcelona, Barcelona, Spain; 7 Group of Dental and Maxillofacial Pathology and Therapeutics, IDIBELL Research Institute, Barcelona, Spain; 8 Department of Dental Medicine, Karolinska Institute, Stockholm, Sweden; 9 Faculty of Dentistry, University of Hong Kong, Pok Fu Lam, Hong Kong, China

**Keywords:** Short dental implants, Dental implants, Bone regeneration, Atrophic mandible, Cost-Effectiveness Analysis

## Abstract

**Background:**

To evaluate the cost-effectiveness of short implants (6–8.5 mm) placed without guided bone regeneration (GBR) vs. regular length implants (> 10 mm) placed with vertical GBR for the rehabilitation of the posterior atrophic mandible (Seibert type II defects).

**Methods:**

A discrete-time state-transition microsimulation model was developed to compare two strategies over an 8-year horizon from a private-payer perspective. Clinical inputs were extracted from a recent meta-analysis of randomized trials, while unit costs were obtained from publicly available Chilean fee schedules and converted to 2025 USD using purchasing power parity. Outcomes included implant survival, biological and prosthetic complications, summarized as implant-years (IY) and complication-free IY (CFIY) as a stricter secondary measure. Incremental cost-effectiveness ratios (ICERs) were calculated. Uncertainty was addressed through one-way deterministic sensitivity analysis (OWSA), and probabilistic sensitivity analysis (PSA, 2,000 iterations).

**Results:**

Over an 8-year horizon, short implants were associated with lower mean costs (USD 3,662 vs. 6,030) and modestly greater effectiveness (6.77 vs. 6.49 IY; 6.71 vs. 6.27 CFIY) compared with regular-length implants with GBR, yielding incremental savings of USD 2,367 and incremental gains of 0.28 IY and 0.44 CFIY. OWSA identified initial implant costs as the most influential parameters. PSA across 2,000 iterations corroborated these findings, with the great majority of simulations falling in the south-east quadrant of the cost-effectiveness plane.

**Conclusions:**

Within the Chilean private-payer setting and over an 8-year horizon, single-tooth short implants (6–8.5 mm) placed without GBR were cost-saving and at least non-inferior in effectiveness compared with regular-length implants with vertical GBR for the posterior atrophic mandible. This cost advantage remained consistent across deterministic, probabilistic, and scenario analyses, although transferability to other settings requires re-estimation using local cost structures.

**Supplementary Information:**

The online version contains supplementary material available at 10.1186/s12903-026-09152-2.

## Background

The rehabilitation of the posterior atrophic mandible with implant-supported prostheses poses a challenge that demands a high level of surgical skill and expertise to ensure proper technique execution. The anatomical complexity of the surgical site should be carefully considered, particularly its proximity to the inferior alveolar nerve and adjacent structures such as the floor of the mouth and sublingual gland. Attention should be also paid to muscle insertions (buccinator and mylohyoid), cortical density and bone quality [1].

Patients with Seibert Class II classification atrophy [2], characterized by adequate bone width but deficient vertical height, are candidates for two treatment approaches. They may undergo reconstructive procedures to augment bone volume prior to placement of standard-length implants, or alternatively short implants can be placed in the residual native bone. At present, one of the most accepted therapeutic strategies for the atrophic mandible involves the placement of regular or standard-length endosseous implants after bone augmentation or simultaneously. Although generally effective, reconstructive procedures are associated with a notable risk of complications: 6.8–57.1% in distraction osteogenesis, 2.5–100% with bone block grafts, and 5.8–27.3% for guided bone regeneration (GBR) [3, 4], and require several extra months of healing to deliver the prosthesis.

As an alternative, the use of short implants in the atrophic alveolar ridge avoids the frequent morbidity and prolonged treatment time associated with bone augmentation. A recent systematic review and meta-analysis, which synthesized randomized controlled trials (RCTs) with long-term follow-up, demonstrated that short implants placed in native bone achieved comparable survival rates to regular implants combined with GBR, while showing lower biological complication rates and reduced marginal bone loss [5]. However, to date, an economic evaluation of short implants has not been reported in the literature. From the patient’s perspective, cost reduction is often a decisive factor in treatment choice [6].

This study aimed to assess the cost-effectiveness of short implants without GBR compared with regular length implants with vertical GBR in the posterior atrophic mandible. We hypothesize that short implants represent a more cost-effective strategy compared to regular length implants with GBR in this clinical setting.

## Methods

The current study is a model-based full economic evaluation. The reporting of the economic evaluation complies with the Consolidated (CHEERS 2022) checklist [7] and was developed following the ECOBIAS checklist [8].

### Study population and setting

The target population was partially edentulous adults requiring single-tooth replacement in the posterior mandible. We adopted the private-payer perspective because short implant therapy is not currently covered in the Chilean public health system, where implant treatment is restricted to specific programs [9]. Consequently, the relevant decision context for patients and clinicians remains the private sector, where treatment is provided. Additionally, cost data for dental implants are publicly available from private providers (Available in: https://www.integramedica.cl/integramedica/site/docs/20180212/20180212164438/arancel_dental_2025.pdf), ensuring transparency and reproducibility of the analysis. Finally, the private payer perspective captures the real-world economic burden on patients, which is particularly relevant in Chile, where out-of-pocket expenditure accounts for a substantial share of oral healthcare financing [10].

### Interventions

The clinical protocols adopted in this study were based on the synthesis provided by a recent systematic review [5]. Surgical protocols involved flap elevation, minimum insertion torque of 25 Ncm, and healing times of 4–5 months for grafted sites. Antibiotic prophylaxis was commonly reported in augmented groups, less consistently in the short-implant groups. Prosthetic rehabilitation followed a conventional staged approach: provisional loading at 4 months and definitive restoration at 8 months (metal–ceramic, metal–resin, zirconia, or titanium–resin prostheses), with either screw-retained or cemented retention. Risk factors such as smoking, bruxism, and history of periodontitis were variably present across studies. In this sense, two interventions were modeled for scheduled-fee resource-based costing [11]:

- Single-tooth short implant (6–8.5 mm) placed in the posterior atrophic mandible with adequate buccolingual bone width, without vertical bone augmentation.
- Single-tooth regular length implant (> 10 mm; e.g., 4.25 × 11.5 mm) placed in the posterior atrophic mandible with simultaneous vertical bone augmentation, including implant placement, GBR procedure with collagen membrane, particulate bone graft (xenogeneic or allogeneic, 2 cc) and fixation (pins or screws).

### Time horizon and discounting

A base-case horizon of 8 years was adopted, capturing the longest follow-up reported within the 6–8.5 mm subgroup of the source meta-analysis, which allows the model to remain anchored to empirical data rather than to extrapolation [5]. Both costs and outcomes were discounted at an annual rate of 3%, consistent with international reference-case guidance for cost-effectiveness analyses [12]. The rate varied between 0 and 5% in the OWSA [13].

### Outcomes

The primary outcome was implant survival, defined as the implant remaining in situ for the duration of the study period. Secondary outcomes included biological complications, defined as the occurrence of mucositis or peri-implantitis, and prosthetic complications, defined as screw loosening, decementation, ceramic chipping, prosthesis fracture, or crown replacement. From these outcomes we constructed the effectiveness measure “implant-years” (IY) defined as the time the implant remained fully functional (not failed). Data for these outcomes were extracted from a recent meta-analysis of studies conducted in private dental practice settings, restricted to the 6–8.5 mm short-implant subgroup to ensure a clinically and biomechanically homogeneous comparator and to avoid the substantial between-subgroup heterogeneity for biological complications observed when ultra-short (4- < 6 mm) and short (6–8.5 mm) implants are pooled (test for subgroup differences *p* < 0.01) (Table 1) [13]. Survival curves for each arm were generated using microsimulation (20,000 traces per iteration) and summarized across 2,000 probabilistic iterations. Median survival estimates and 95% uncertainty intervals (UIs) were calculated at each cycle and presented graphically.

**Table 1:** Event counts

Time (year)	Short implants (6–8.5 mm)			Regular-length implants with guided bone regeneration		
	Failure	Biological complication	Prosthetic complication	Failure	Biological complication	Prosthetic complication
1	6/187	3/187	0/43	19/192	36/192	0/42
3	2/101	7/101	3/43	6/108	35/108	7/42
5	8/147	11/147	11/62	7/159	55/159	12/59
8	5/60	9/60	4/25	3/61	27/61	3/23

Numerators are events; denominators are implants at risk pooled across contributing trials within each follow-up cross-section. Year-1 prosthetic denominators are taken from the year-3 reporting cross-section, since the meta-analysis did not report prosthetic outcomes earlier than three years post-loading; the assumed year-1 event count of 0 is consistent with the absence of prosthetic complications reported during the first year of function in the source RCTs. Year-8 estimates derive from a single trial. Reductions in denominators between cross-sections reflect study attrition and differential study contribution at each time point rather than within-cohort losses to follow-up

### Measurement and valuation of resources and costs

A scheduled-fee resource-based costing exercise was conducted using direct unit costs originally obtained in Chilean pesos (CLP) from publicly available private fee schedules. To ensure international comparability, costs were converted into US dollars (USD) using the 2025 purchasing power parity (PPP) exchange rate instead of the nominal exchange rate. PPP was chosen because it reflects the relative price level of goods and services across countries and avoids distortions caused by currency fluctuations. According to International Monetary Fund data, the PPP conversion factor for Chile in 2025 was approximately CLP 464.99 per USD (PPP) [14]. Unit costs were therefore divided by this factor to obtain their equivalent values in USD (Table 2). The costing strategy was validated by experts in the field (MB, SF). In addition, the cost of a prosthetic complication was incorporated as an empirical frequency-weighted average derived directly from the prosthetic events reported in the trials contributing prosthetic complication data to the source meta-analysis screw loosening/retightening (30.8%), recementation (23.1%), and ceramic chipping or simple prosthetic repair (46.2%) [13], yielding a base-case cost of USD 189 per event. Higher-cost prosthetic interventions not observed in the pooled event distribution were retained as conservative scenario analyses (Table 2).

**Table 2:** Unit costs used as model parameters (Chilean fee schedule, calculated 25 September 2025; converted to 2025 USD using the IMF purchasing power parity factor of 464.99 CLP per USD-PPP applied uniformly across both arms)

Item	Code (fees chart)	Cost (CLP 2025)	Cost (USD 2025)
*Intervention*			
Implant placement (professional fees)	6201005	504,489	1,085
Short implant (unit)	50030171	424,474	913
Single implant surgery room	6401007	87,550	188
Healing abutment	50022619	47,828	103
Prosthetic connection	6201014	71,847	154
Crown on implant (CAD/CAM)	6004038	531,041	1,142
Total cost		**1,667,229 **	**3,586**
*Comparator*			
Implant placement (professional fees)	6201005	504,489	1,085
Regular length implant (unit)	50031773	181,448	390
Single implant surgery room	6401007	87,550	188
GBR (procedure)	6201008	249,902	537
Collagen membrane 20 × 30	50007073	295,936	636
Particulate bone graft 2 cc (xeno/human)	50009517	682,628	1,468
Fixation (screw/pin)	50032326	62,087	134
Healing abutment	50022619	47,828	103
Prosthetic connection	6201014	71,847	154
Crown on implant (CAD/CAM)	6004038	531,041	1,142
Total cost		**2,714,756**	**5,838**
Biological complication (conservative surgical management)	6007039	156,189	336
Prosthetic complication (weighted average)^1^		**87,776**	**188.77 **

^1^Prosthetic complication cost was estimated as a frequency-weighted average using the prosthetic events directly reported in literature: screw loosening/retightening (30.8%; codes 50022099 plus clinical visit, USD 138), recementation of a cemented crown or fixed partial denture (23.1%; code 6004001, USD 202), and ceramic or prosthetic fracture requiring simple prosthesis repair (46.2%; code 2706005, USD 216). Bold indicates the total costs

### Statistical analysis

#### Model structure

**Fig. 1:**
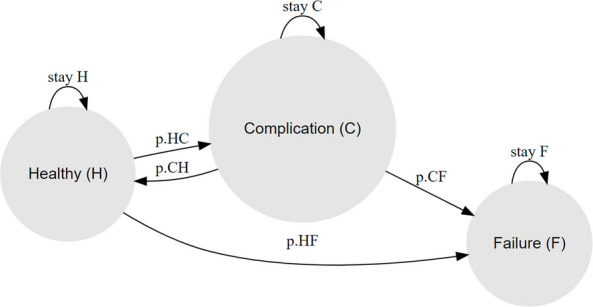
Schematic representation of the microsimulation model. Implants enter the model in the functional state. In each cycle, implants may remain functional, transition to a biological complication state, or fail. Implants in the complication state may return to the functional state (with an event-related disutility period) or transition to failure. Failure is an absorbing state. In addition, an independent prosthetic-event hazard is applied at every cycle to all implants in states H or C

The model was implemented as a time-inhomogeneous (discrete-time) Markov state-transition microsimulation, with cycle-specific transition probabilities and three health states: functional implant ($H$), complication ($C$), and failure ($F$) (Fig. 1). Transition probabilities vary over time (by cycle/interval). However, the model does not include explicit patient-level event-history memory (e.g., prior complication count, complication subtype/severity, or time since the last complication episode). Thus, repeated transitions between the functional and complication states may occur across cycles, but these are represented as visits to an aggregated complication state rather than as fully tracked individual recurrent trajectories. The model was run over an 8-year horizon, with 0.5-year cycles and an annual discount rate of 3%. Input parameters are detailed in Table 3. This model was preferred over a discrete-event-simulation or semi-Markov framework because the available evidence base reports event counts at fixed follow-up cross-Sects. (1, 3, 5, and 8 years) rather than individual event-time distributions, making piecewise-constant interval hazards across discrete cycles the most defensible representation of the empirical data [15].

**Table 3:** Parameters imputed in the microsimulation model

Input parameter	Value	Distribution	Parameters	Source/Notes
Initial cost – Short implant	3585.52 (USD 2025)	Gamma	mean = μ; CV = 0.10	Fee schedule PPP-adjusted
Initial cost – Regular + GBR	5838.31 (USD 2025)	Gamma	mean = μ; CV = 0.10	
Complication cost – Short	335.90 (USD 2025)	Gamma	mean = μ; CV = 0.20	
Complication cost – Regular + GBR	335.90 (USD 2025)	Gamma	mean = μ; CV = 0.20	
Failure cost – Short	0 (absorbent state)	Fixed (tested in PSA)	Gamma distribution, mean = μ; CV = 0.20	Tested in sensitivity analysis
Failure cost – Regular + GBR	0 (absorbent state)	Fixed (tested in PSA)	Gamma distribution, mean = μ; CV = 0.20	
Event-related downtime at complication onset	30 days	Beta (re-scaled, bounded)	mean = μ; Bounds = 15–90 days; CV = 0.15–0.25	Expert panel
Transition probabilities	Event counts from Table 1	Beta resampling from event counts	Continuity-corrected pseudo-counts (0.5) for PSA	Meta-analysis
Discount rate	3%	Fixed	0–5% for OWSA	Literature

μ indicate that the parameter used was showed in the “Value” column

*CV* Coefficient of variation, *OWSA* One-way sensitivity analysis, *PSA* Probabilistic sensitivity analysis

#### Transition probabilities and hazard calibration

For each arm, transition matrices ${P}_{k}$​ were defined for every cycle $k$ by converting piecewise-constant hazards $\lambda$ into transition probabilities under a competing-risks framework. Hazards were estimated from crude event counts at 1, 3, 5, and 8 years, which were first transformed into cumulative hazards $H\left(t\right)=-\mathrm{ln}(1-e/n)$. Interval-specific hazards were then obtained as differences between adjacent cumulative hazards, yielding a piecewise-constant approximation. In this way, the hazard structure of the model was calibrated over time, with higher hazards applied in earlier intervals if suggested by the empirical data. Because the cumulative incidence at each follow-up cross-section was estimated from partly non-overlapping sets of trials, isolated negative interval hazards may arise as a sampling artifact of cross-sectional aggregation rather than reflecting biological recovery; these were clipped to zero. Interval hazards in this framework therefore describe the expected behavior of a synthetic average implant under the meta-analytic evidence base, rather than the trajectory of any single cohort.

#### Biological complications and risk adjustments

Exponential recovery from the $C$ state (${\lambda}_{CH}=365/{d}_{days}$​) and a multiplier for increased failure risk after $C$ (${\lambda}_{CF}={k}_{CF}\cdot {\lambda}_{HF}$​) were also incorporated. The complication-state recovery parameter (${d}_{days}$) was fixed at 30 days in the base case for transition modeling, and an event-related effectiveness decrement (downtime) at complication onset ($H\to C$) was also fixed at 30 days in the base case. The multiplier for failure risk after complication was assumed to be 1.5 in the base case (varied between 1.0 and 3.0 in sensitivity analysis), informed by recent comparative survival data showing that implants experiencing biological complications retain a median time-in-function of 91.1 months when supportive (rescue) therapy is delivered, compared to 40.9 months without [16]. It was intended to reflect the clinically plausible increase in downstream failure risk associated with peri-implant inflammatory complications and related patient-level risk modifiers [17].

#### Prosthetic complications

Prosthetic complications were modelled as recurrent point events occurring independently of the biological state, since events such as screw loosening, decementation, ceramic chipping, or crown replacement do not by themselves alter the peri-implant tissue health status. For each cycle, an arm-specific prosthetic event hazard derived from the meta-analytic counts (Table 1) was applied to all implants not in the failure state; when an event occurred, a unit cost (Table 2) and a 14-day downtime were added, but the biological state was not changed. This formulation preserves the three-state structure of the Markov model while explicitly accounting for prosthetic resource use and effectiveness loss in both arms, and enables a threshold analysis on the differential prosthetic hazard between arms.

#### Effectiveness measure

Individual trajectories were simulated by sequentially sampling next states from ${P}_{k}$​, generating stochastic realizations of time in each state. Effectiveness was measured in IY, defined as discounted survival time outside failure: $IY=\sum_{k=1}^{K}1\left\{{X}_{k}\ne F\right\}\Delta t{(1+r)}^{-(k-1/2)\Delta t}$. Internal validity was checked by comparing simulated survival proportion curves with those reported in the source RCTs, showing consistent alignment [5]. As a secondary effectiveness measure, complication-free implant-years (CFIY) were also computed by accumulating discounted time spent in the functional state alone (i.e., excluding time spent in the complication state). CFIY was reported alongside IY to address the limitation that survival-time metrics may not adequately reflect the burden of recurrent biological events, reporting both metrics allowed assessment of whether the cost-effectiveness ranking is robust to a stricter, complication-aware definition of effectiveness.

#### Costing

Costs included an initial intervention cost ${C}_{0}$​ and event-related costs ${c}_{comp}$​, ${c}_{fail}$, all discounted at the mid-cycle point ($Cost={C}_{o}+\sum_{k=1}^{K}\left[{c}_{comp}1\left\{H\to C\right\}+{c}_{fail}1\left\{* \to F\right\}\right]$).

#### Simulation and outcomes

Across $N$=100.000 simulated patients per arm in the base-case, expected outcomes ($\overline{IY },\overline{C })$ were obtained, and incremental values computed as $\Delta IY={\overline{IY} }_{short}-{\overline{IY} }_{regular}$ and $\Delta C={\overline{C} }_{short}-{\overline{C} }_{regular}$. The incremental cost-effectiveness ratio (ICER) was then defined as $ICER=\frac{\Delta C}{\Delta IY}$. Mean, standard deviation and Montecarlo standard error (MCSE) was reported.

#### Deterministic sensitivity analysis (OWSA)

We conducted a one-way deterministic sensitivity analysis (OWSA) varying key cost inputs by ± 20%, the discount rate from 0 to 5%, the failure multiplier from the functional state (range 0.75–1.25), the failure-multiplier post-complication kCF (range 1.0–3.0), the biological event-related downtime (15–90 days), the prosthetic event-related downtime (7–30 days), and the prosthetic-event cost (USD 138–469, covering the full range from least-costly screw retightening to highest-cost routine prosthetic intervention), while holding other parameters constant. For each scenario, we re-ran the microsimulation ($N$=100.000 simulated patients per arm) and computed the incremental net monetary benefit (INMB) at a fixed WTP of 500 USD per implant-year. Results are presented as a tornado plot ordered by the impact on INMB.

#### Probabilistic sensitivity analysis (PSA)

Uncertainty was addressed through a probabilistic sensitivity analysis (PSA) with 2,000 iterations and 20,000 Monte Carlo microsimulated patients per arm per iteration. Event probabilities were resampled from Beta distributions using continuity-corrected pseudo-counts based on observed event counts and denominators, while costs were drawn from Gamma distributions with parameters matched to mean and coefficient of variation (CV). For initial costs, a base CV of 0.10 was used, and for event costs a base CV of 0.20. In addition, random CV values were drawn in each iteration within plausible ranges (uniform distribution between 0.15 and 0.25) to reflect second-order uncertainty in economic inputs (beyond the first-order sampling of clinical events). The event-related downtime parameter (base-case 30 days) was modeled probabilistically using a Beta distribution re-scaled to a bounded range of 15–90 days, with iteration-specific CVs sampled from 0.15 to 0.25 (applied on the day scale), as a pragmatic uncertainty representation informed by expert elicitation, given the heterogeneity of peri-implantitis treatment modalities and the limited/inconsistent reporting of recurrence, retreatment, and timing of additional interventions in the published literature [18]. The structural transition framework (including complication-state occupancy) was kept fixed in PSA, while the event-related burden parameter was varied probabilistically. For each iteration, total discounted costs and effectiveness (implant-years) were estimated for both strategies, and incremental costs, incremental effectiveness, and ICERs were computed. PSA results were summarized using descriptive statistics and displayed on the cost-effectiveness plane. We additionally summarized the probability of cost-effectiveness at a conservative WTP threshold of USD 500 per implant-year (IY). This corresponds to approximately one-third of the WTP values reported in implant-therapy studies [19, 20] and to about half of Chile’s monthly minimum wage (CLP 529,000, 2025), after conversion to PPP-adjusted USD (USD 1,137.66). All the analysis was conducted using R version 4.5.1 (https://www.R-project.org/).

#### Scenario analyses

To address structural uncertainty, pre-specified scenarios re-ran the base-case microsimulation under: (i) a 5-year horizon (anchored to the most evidence-dense follow-up window of the source meta-analysis); (ii) a conservative kCF of 1.0 (no downstream amplification of failure hazard following biological complication); (iii) the original expert-opinion kCF of 2.0; (iv) a worst-plausible kCF of 3.0; (v) exclusion of the prosthetic-complication module from both arms; and (vi) a worst-case prosthetic-event cost of USD 1,148, corresponding to crown replacement applied to every prosthetic event. Each scenario reports incremental costs, IY, CFIY, and ICER. In addition, to specifically address whether unobserved excess prosthetic maintenance in the short-implant arm could reverse the conclusion, we performed an adverse prosthetic-hazard threshold analysis. In this analysis, the prosthetic-event hazard for short implants was progressively multiplied while keeping all other parameters unchanged. The threshold was defined as the multiplier at which INMB crossed zero.

## Results

In the base-case analysis, mean costs were lower for short implants (USD 3,662) compared with regular length implants with GBR (USD 6,030), yielding an incremental cost saving of USD 2,367 over the 8-year horizon (Table 4). Monte Carlo standard errors were negligible (< USD 0.05), confirming the stability of these estimates.

**Table 4:** Results obtained from the microsimulation model

Scenario	Short implants			Regular length implants and guided bone regeneration			$\Delta$ Costs	$\Delta$ Effectiveness (IY)	$\Delta$ Effectiveness (CFIY)
	Cost (USD 2025)	IY	CFIY	Cost (USD 2025)	IY	CFIY			
Base case	3662.17	6.77	6.71	6029.54	6.49	6.27	−2367.37	0.28	0.44
PSA	3689.51 (SD 687.38)	6.73 (SD 0.09)	6.66 (SD 0.09)	6052.72 (SD 1120.78)	6.45 (SD 0.13)	6.22 (SD 0.13)	−2363.21 (SD 1307.20)	0.28 (SD 0.16)	0.44 (SD 0.16)

*CFIY* Complication-free Implant Years, *ICER* Incremental cost-effectiveness ratio, *IY* Implant Years, *PSA* Probabilistic sensitivity analysis, *MCSE* Montecarlo standard error, *SD* Standard deviation

**Fig. 2:**
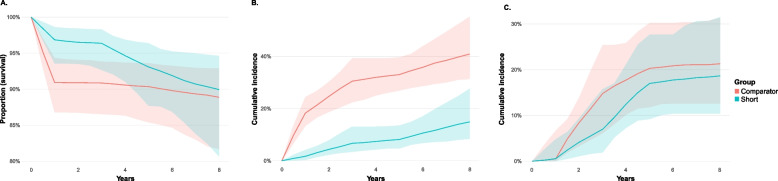
Survival and cumulative-incidence curves over the 8-year horizon, by treatment arm. **A** Model-predicted implant survival, defined as the proportion of implants remaining functional. **B** Model-predicted cumulative incidence of the first biological complication. **C** Model-predicted cumulative incidence of the first prosthetic complication

In terms of effectiveness, short implants yielded a modestly higher mean of 6.77 IY versus 6.49 for regular length implants with GBR, resulting in an incremental gain of 0.28 implant-years (Table 4). When effectiveness was measured in CFIY, the incremental gain widened to 0.44 CFIY (6.71 vs. 6.27), reflecting the lower biological-complication burden in the short-implant arm. Because short implants were both less costly and at least non-inferior in effectiveness across both metrics, they were located in the cost-saving quadrant relative to the comparator under either definition (ICERs: -USD 8,455 per IY; -USD 5,380 per CFIY). Figure 2a shows the survival curves over the 8-year horizon, defined as the proportion of implants remaining functional (healthy or complicated states). Both strategies maintained high survival probabilities (> 0.90 at 5 years; > 0.85 at 8 years), with partially overlapping curves; the model-predicted cumulative incidence of biological and prosthetic complications (Fig. 2b and 2 C) was in close agreement with the four meta-analytic time points used as model inputs (1, 3, 5, and 8 years), supporting the internal validity of the calibration.

**Fig. 3:**
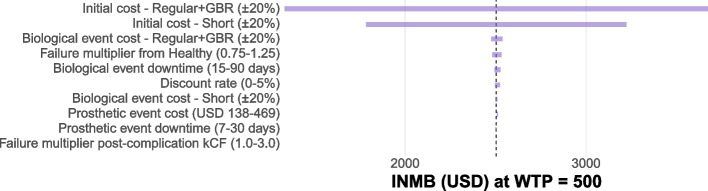
Tornado diagram from the one-way deterministic sensitivity analysis (OWSA), showing the impact of varying key model parameters on the incremental net monetary benefit (INMB) at a WTP of 500 USD per implant-year

Figure 3 presents the tornado diagram from the one-way sensitivity analysis, illustrating the impact of varying individual model parameters on the incremental net monetary benefit (INMB) at a willingness-to-pay threshold of USD 500 per implant-year. The parameters with the greatest influence were the initial cost of the comparator (regular length implant with GBR) and the initial cost of short implants (each varied by ± 20%), which generated the widest ranges of INMB around the base-case estimate. The remaining parameters, biological and prosthetic event costs, the discount rate (0–5%), the failure multiplier from the functional state (0.75–1.25), the failure-multiplier post-complication kCF (1.0–3.0), the biological event-related downtime (15–90 days), the prosthetic event-related downtime (7–30 days), and the prosthetic-event cost (USD 138–469), had comparatively limited effects on INMB. Importantly, INMB remained positive across all OWSA bounds, including the upper bound of kCF and the upper bound of prosthetic-event cost.

**Fig. 4:**
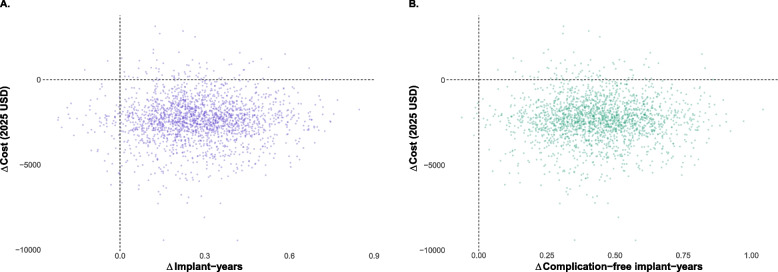
Cost-effectiveness plane from probabilistic sensitivity analysis (2,000 iterations). Each point represents one PSA simulation, plotted as incremental cost against incremental effectiveness, for short implants relative to regular-length implants with GBR. **A** ΔIY. **B** ΔCFIY). Most simulations fall in the south-east quadrant (lower cost, greater effectiveness) under both metrics

The PSA was directionally consistent with the base case. Across 2,000 iterations, short implants showed lower mean costs (USD 3,690 vs. USD 6,053) and modestly higher effectiveness (6.73 vs. 6.45 IY; 6.66 vs. 6.22 CFIY), with incremental savings of USD 2,363 (SD≈1,307) and incremental effectiveness of 0.28 IY (SD≈0.16) and 0.44 CFIY (SD≈0.16) (Table 4). Figure 4 presents the cost-effectiveness plane on both metrics, where the great majority of simulated pairs fell in the south-east quadrant, indicating that most simulations favored short implants as the less costly strategy with slightly greater effectiveness under either outcome definition at the fixed WTP (96.9% and 97.1% of probability of being cost-effective for IY and CFIY, respectively).

Across the scenario analyses (Table 5), the cost advantage of short implants was preserved in all cases. Restricting the model to a 5-year horizon, anchored to the most evidence-dense follow-up window of the source meta-analysis, yielded an incremental cost saving consistent in direction with the 8-year base case. Varying kCF over its full plausible range shows that cost advantage is held. Excluding the prosthetic-complication module from both arms slightly increased the incremental cost saving, indicating that prosthetic complications, while present in both arms, do not significantly drive the result. A worst-case prosthetic-event cost (USD 1,148, equivalent to assuming every prosthetic event requires crown replacement) reduced the incremental cost saving by less than 10%. Effectiveness rankings (IY and CFIY) likewise favored short implants in all scenarios. The adverse prosthetic-hazard threshold analysis showed that increasing the prosthetic-event hazard in the short-implant arm progressively reduced INMB. However, INMB remained positive across the full explored range and did not cross zero, even when the short-implant prosthetic-event hazard was multiplied by 20 (Supplementary Figure S1).

**Table 5:** Scenario analysis

Scenario	Cost Comparator	Cost Intervention	$\Delta$ Costs	$\Delta$ IY	$\Delta$ CFIY	ICER_IY_	ICER_CFIY_
Conservative kCF = 1.0	6030.05	3661.50	−2368.56	0.25	0.41	−9553.68	−5806.22
kCF = 2.0 (original assumption)	6029.84	3661.58	−2368.26	0.26	0.42	−9255.48	−5694.56
kCF = 3.0 (worst plausible)	6030.00	3661.51	−2368.50	0.26	0.42	−9207.56	−5671.68
5-year time horizon	5990.81	3640.52	−2350.29	0.23	0.37	−10,100.06	−6389.79
Without prosthetic events	5994.57	3630.49	−2364.08	0.29	0.45	−8204.79	−5282.28
High prosthetic costs (USD 469)	6082.99	3709.10	−2373.89	0.25	0.41	−9433.70	−5768.71
Worst-case prosthetic costs (USD 1,148, replacement)	6211.94	3824.34	−2387.61	0.25	0.41	−9488.20	−5802.04

## Discussion

This is the first exploratory economic evaluation which finds, within the Chilean private-payer perspective and over an 8-year horizon, that single-tooth short implants (6–8.5 mm) placed in native bone are cost-saving and at least non-inferior in effectiveness compared with regular length implants placed with vertical GBR for the rehabilitation of the posterior atrophic mandible. Within this context, short implants were positioned as a cost-saving strategy with slightly higher modelled effectiveness than the comparator. This economic ranking, however, should be interpreted as setting-specific rather than universal, since the cost advantage is driven primarily by the avoidance of bone-substitute and barrier-membrane materials whose unit prices vary substantially across health systems. This finding supports considering short implants as a less invasive and potentially more cost-effective option when both strategies are clinically feasible and local cost structures resemble those modelled here. In the context of the totality of evidence, our results reinforce findings from systematic reviews indicating no significant differences in survival between short implants without GBR and longer implants with GBR [5, 21]. By adding an economic perspective, this study addresses a key gap in the literature: while clinical equivalence has been established, the financial impact of avoiding augmentation procedures has not been quantified. Our results provide economic evidence that may support shared decision-making in favor of short implants when augmentation is otherwise required and the residual ridge width is adequate [5], particularly in middle-income countries where out-of-pocket payments remain a major barrier to oral rehabilitation [22]. However, these findings are limited to cases with Seibert type II defects, e.g. atrophy with enough ridge width to place implants. In these cases, regular length implants can only be placed with 2 groups of surgical techniques: vertical augmentation (either with bone grafts or osseous distraction) [4, 23] or lateralization of the inferior alveolar nerve [24]. Both techniques carry higher surgical risks of postoperative complications and morbidity, the former one requiring a significantly longer healing time. Other situations, such as narrow ridges, are out of the scope of our study and require further investigation.

Our study found a modest difference of 0.28 implant-years favoring short implants over regular implants with GBR (6.77 vs. 6.49 over the 8-year horizon). This small difference should be interpreted cautiously. It may reflect event patterns observed in the source trials rather than a definitive clinical superiority of short implants. Nevertheless, biomechanical studies provide a plausible rationale for the clinical viability of short implants in selected cases, where reliability, stress distribution, and primary stability have been systematically evaluated. An in vitro study demonstrated that 5-mm extra-short implants supporting single crowns or fixed partial dentures reached reliability above 84% under 200 N, with greater performance at wider diameters and improved stress distribution when splinting was applied [25]. Finite element analyses further showed that short implants can generate lower von Mises stresses than longer implants, particularly when diameter is optimized, supporting their potential substitution in the native mandible [26]. Functional load on narrow implants is distributed over a smaller surface area, resulting in higher loads that can lead to accelerated bone remodeling and loss [27]. Remaining controversies include their long-term performance under high occlusal loads or in patients with poor bone quality [28, 29], although previous synthesis suggests comparable outcomes under these conditions [5].

Several strengths of this work enhance its validity. The model was informed by a recent systematic review and meta-analysis of randomized trials, ensuring high-quality clinical evidence [5]. The use of a discrete-time microsimulation framework allowed incorporation of uncertainty through both PSA and OWSA. Limitations include reliance on cost data derived from the Chilean private sector, which may limit generalizability. Results may differ from a public payer perspective, or other countries, where treatment coverage and cost structures vary. The lack of quality-adjusted survival or patient-reported outcomes may undervalue broader benefits. In this sense, it has been observed that patients who receive short implants have greater satisfaction according to the OHIP-14 scale [30]. In addition, the source comparative synthesis did not show clear between-group differences in prosthetic complications/failures across available follow-up periods, with very low certainty of the evidence for this outcome [5]. In this sense, these complications were explicitly modelled and stress-tested because variation in prosthetic design, retention type, materials, occlusal loading, and maintenance patterns may materially influence long-term value estimates [5, 31]. Current evidence indicates that crown-to-implant ratio does not significantly affect implant survival, although it may be associated with increased marginal bone loss [32, 33]. Marginal bone loss and bone remodeling may, over time, increase the risk of implant failure [34]. Therefore, their potential indirect effect through peri-implant bone changes should be acknowledged in the interpretation of our results as they may influence long-term value estimates and should be prioritized in future models with richer longitudinal and utility data. Additionally, GBR procedures are heterogeneous in clinical practice, which may introduce variability not fully captured by the model [3, 4]. The base-case 8-year horizon was anchored to the longest follow-up reported within the 6–8.5 mm subgroup of the source meta-analysis, and a 5-year structural scenario showed that the cost-effectiveness conclusion did not depend on this choice. Extrapolation beyond 8 years was not attempted because it would require parametric assumptions about late failure, biological complication, prosthetic maintenance, and replacement hazards that the available evidence base cannot uniquely identify. This remains an important limitation, particularly in implant dentistry, where long-term maintenance and survival beyond 10 years are clinically relevant [35]. Potential bias could arise from contextual differences in cost structures, patient selection [36, 39] or surgical experience [40, 43]. Finally, the simplified model structure may not fully capture the complexity of real-world implant pathways, including recurrent complications, staged retreatment, prosthetic replacement, patient preferences, and quality-of-life effects. More granular models using patient-level longitudinal data are needed to determine whether these omitted pathways would attenuate or amplify the observed cost difference.

In terms of clinical and policy implications, short implants offer tangible advantages for patients and health systems. For individuals, reduced treatment cost and morbidity translate into lower barriers to care. For providers and policymakers, these findings suggest that short implants may be worth considering as a resource-sparing alternative in selected cases, particularly where both treatment strategies are clinically feasible and augmentation-related costs are borne directly by patients.

## Conclusion

Within the Chilean private-payer setting and over an empirically anchored 8-year horizon, single-tooth short implants (6–8.5 mm) placed without GBR were cost-saving and at least non-inferior in modelled effectiveness compared with regular-length implants placed with vertical GBR for rehabilitation of the posterior atrophic mandible, particularly when ridge width is preserved and the limitation is confined to vertical bone height. The cost advantage was driven primarily by avoiding bone-substitute and barrier-membrane materials and remained consistent across sensitivity and scenario analyses, including prosthetic-event assumptions. These findings support considering short implants as a less invasive and potentially cost-saving option in this specific clinical and economic context, while emphasizing that transferability to other settings requires re-estimation using local costs, longer-term evidence, and broader patient-centered outcomes.

## Supplementary Information

Supplementary Material 1.

## Data Availability

The datasets used and/or analyzed during the current study are available from the corresponding author on reasonable request.
