# Supplementary figures and images for: Cost-effectiveness of short implants (6–8.5 mm) compared to regular length implants (> 10 mm) with bone regeneration in posterior atrophic mandible: a 8-year microsimulation model
Source: BMC Oral Health. 2026 Jul 10;26:1241. doi: 10.1186/s12903-026-09152-2 (PMC13352667; doi:10.1186/s12903-026-09152-2)

**INMB (USD) at WTP=500/implant-year**

2500  
2000  
1500  
1000  
500  
0

5

10

15

20

**Multiplier on short implants prosthetic hazard**

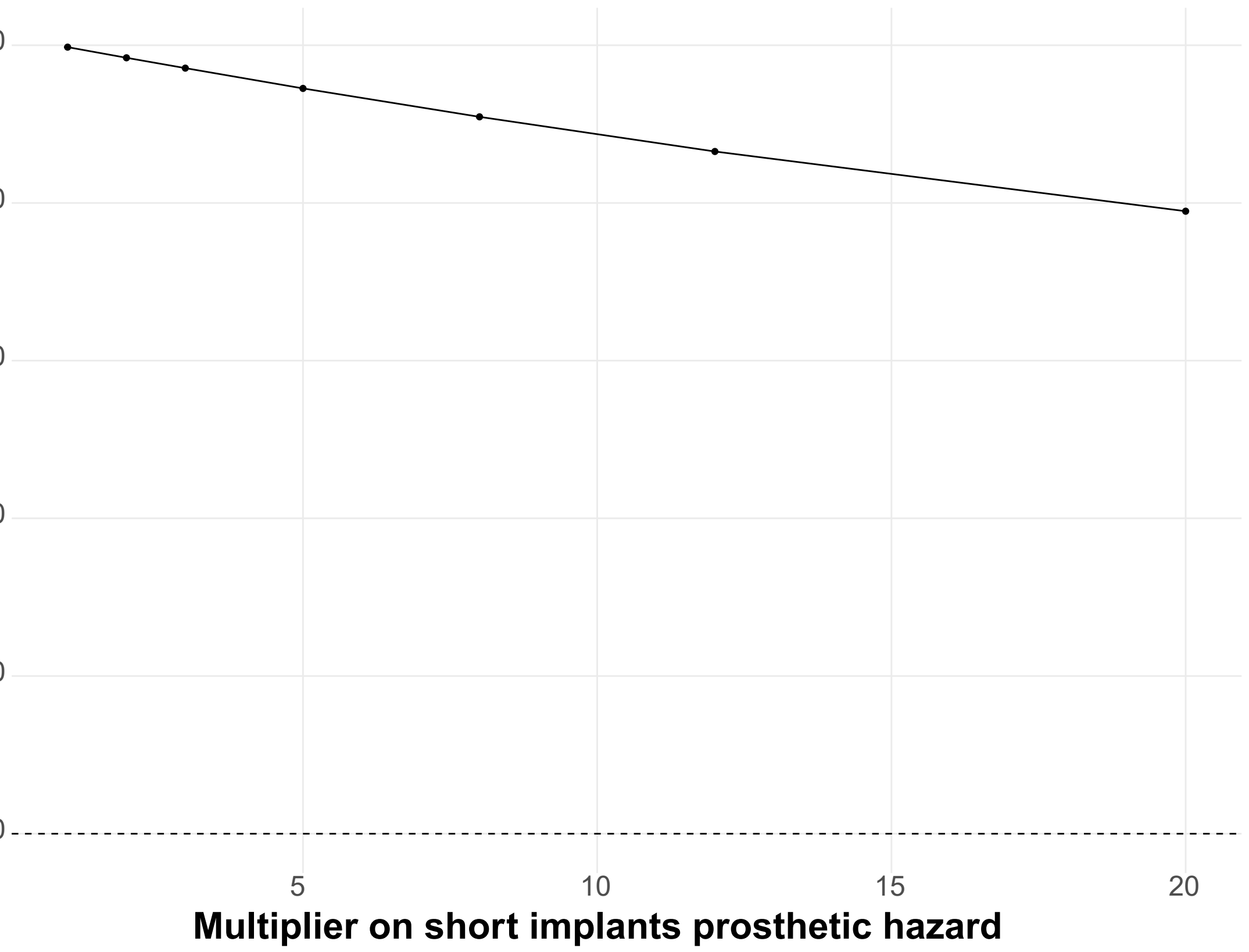

Supplement: Supplementary file 1 — Supplementary Material 1. [file 12903_2026_9152_MOESM1_ESM.pdf]
